# Actin Is a Target of T-Cell Reactivity in Patients with Advanced Carotid Atherosclerotic Plaques

**DOI:** 10.1155/2013/261054

**Published:** 2013-11-11

**Authors:** Elisabetta Profumo, Brigitta Buttari, Linda Petrone, Giada Lacroce, Maria Chiara Tesori, Raffaele Capoano, Bruno Salvati, Rachele Riganò

**Affiliations:** ^1^Department of Infectious, Parasitic and Immune-Mediated Diseases, Istituto Superiore di Sanità, 299 Viale Regina Elena, 00161 Rome, Italy; ^2^Department of Surgical Sciences, Sapienza University of Rome, 155 Viale del Policlinico, 00161 Rome, Italy

## Abstract

Atherosclerosis is a chronic inflammatory disease of the arterial wall associated with autoimmune reactions. In a previous study, we observed the presence of actin-specific antibodies in sera from patients with carotid atherosclerosis. To extend our previous results we evaluated the possible role of actin as antigenic target of cell-mediated immune reactions in carotid atherosclerosis. Peripheral blood mononuclear cells (PBMC) from 17 patients and 16 healthy subjects were tested by cell proliferation assay and by ELISA for cytokine production. Actin induced a proliferative response in 47% of patients' PBMC samples, with SI ranging from 2.6 to 21.1, and in none of the healthy subjects' samples (patients versus healthy subjects, *P* = 0.02). The presence of diabetes in patients was significantly associated with proliferative response to actin (*P* = 0.04). IFN-**γ** and TNF-**α** concentrations were higher in PBMC from patients than in those from healthy subjects and in PBMC proliferating to actin than in nonproliferating ones. Our data demonstrate for the first time a role of actin as a target autoantigen of cellular immune reactions in patients with carotid atherosclerosis. The preferential proinflammatory Th1 activation suggests that actin could contribute to endothelial dysfunction, tissue damage, and systemic inflammation in carotid atherosclerosis.

## 1. Introduction

Atherosclerosis is a chronic inflammatory disease of the arterial wall in which immune responses play a crucial role. Atherosclerotic plaques are characterized by the presence of an inflammatory cell infiltrate mainly composed of macrophages and T lymphocytes that modulate the atherosclerotic process by secreting inflammatory mediators. Infiltrating T lymphocytes are activated T cells expressing CD25 on their surface [1] and predominantly expressing a Th1 phenotype in advanced lesions [2, 3]. In this context, identifying the antigens responsible for T lymphocyte activation in atherosclerosis may be relevant. Accelerated atherosclerosis has been reported in patients with various autoimmune diseases [4–6], suggesting an involvement of autoimmune mechanisms in atherogenesis [7]. Although infectious agents have been associated with the activation of immune mechanisms, several lines of evidence suggest that the main antigenic targets in atherosclerosis are modified endogenous structures [8]. Different self-antigens or modified self-molecules have been identified as target of humoral and cellular immune responses in patients with atherosclerotic disease thus behaving as dangerous signals able to activate proinflammatory responses. Oxidative stress, increasingly reported in these patients [9], is the major event causing structural modification of proteins [10]. 

Oxidized low density lipoproteins (LDL) are the best characterized autoantigen. In particular, it has been demonstrated that about 10% of T lymphocytes infiltrating human atherosclerotic plaques are specific for oxidized LDL [11]. In addition to LDL, other self-molecules modified by oxidative stress become target of autoimmune reaction in atherosclerosis [12–14]. Another two categories of autoantigens that have been implicated in atherosclerosis are the stress-induced heat shock proteins and antigens expressed by dying cells [15, 16]. Cell death in the atherosclerotic plaque may occur by apoptosis or by necrosis [17, 18]. The uptake of apoptotic cells by macrophages and some subsets of dendritic cells may induce an anti-inflammatory response and play an important role in maintaining peripheral immune tolerance [19, 20]. Conversely, the uptake of necrotic cells or even a delayed uptake of apoptotic cells may result in immune activation and risk for the development of autoimmunity [21].

 In a previous study, by the use of a molecular cloning strategy to identify endothelial autoantigens, we provided evidence of serum anti-actin antibodies in patients with carotid atherosclerosis and we suggested that actin is an autoantigenic molecule of potential clinical interest in carotid atherosclerosis [22].

We designed this study to confirm and extend our previous results on the possible role of actin as target antigen of immune reactions in carotid atherosclerosis. For this purpose, we evaluated the proliferative response of circulating T lymphocytes obtained from patients and healthy subjects, stimulated *in vitro* with actin. 

We also investigated the ability of actin-specific circulating T lymphocytes to produce the pro-inflammatory cytokines IFN-*γ* and TNF-*α* and the anti-inflammatory cytokines IL-4 and IL-10. 

## 2. Materials and Methods

### 2.1. Subjects

We enrolled 17 consecutive patients with asymptomatic severe or preocclusive carotid-artery stenosis ≥70% or with symptomatic stenosis undergoing endarterectomy (CEA) at the Sapienza University of Rome. Patients were grouped according to the histological type of their atherosclerotic plaques following the classification of Stary et al. [23]. Thirteen patients had type V plaques and 4 patients had type VI plaques. In brief, type V plaques are defined as lesions in which prominent new fibrous connective tissue has formed. Type VI plaques are defined as lesions in which disruption of the lesion surface, hematoma, or hemorrhage and thrombotic deposits have developed and may be referred to as complicated lesions. The baseline characteristics of patients are reported in Table 1. We also recruited 16 sex- and age-matched healthy subjects as controls. Exclusion criteria for patients were recent infections (<1 month), autoimmune diseases, malignancies, and inflammatory diseases before enrollment. The inclusion criteria for healthy subjects were no history of myocardial infarction, coronary bypass, coronary angiography with angioplasty or stenting or both, cerebrovascular accident, or peripheral vascular disease. None of them had ultrasonographically evident carotid or femoral artery atherosclerotic disease. All hematological variables including risk factors for atherosclerosis were in the range of “normal” values. The investigation conforms with the principles outlined in the Declaration of Helsinki. Informed consent was obtained before enrollment.

### 2.2. Blood Samples

Venous peripheral blood was drawn in heparin tubes from the 17 patients (before surgery) and from the 16 healthy subjects. Peripheral blood mononuclear cells (PBMC) were separated from plasma by density gradient centrifugation (Lympholyte, Cedarlane, ON, Canada) and were used in the proliferation assay. PBMC samples were stored at −80°C until use.

### 2.3. Actin Proliferation Assay

Triplicate cultures of PBMC (1 × 10^6^ cells/mL) were stimulated for 7 days with rabbit muscle actin (Sigma-Aldrich, Milan, Italy, 20 *μ*g/mL), phytohemagglutinin (PHA, Burroughs Wellcome Co., Beckenham, UK, 2 *μ*g/mL) as a positive control of the assay, or human serum albumin (HSA, Sigma-Aldrich, 10 *μ*g/mL) as a negative control, or left unstimulated. Endotoxin contamination in actin, as determined by the quantitative chromogenic Limulus amebocyte lysate assay (QCL-1000, BioWhittaker, Walkersville, MD), was <0.03 endotoxin units/*μ*g of protein. To neutralize a possible endotoxin effect, all cells were cultured in the presence of polymyxin B (10 *μ*g/mL, Sigma-Aldrich).

Cell proliferation was assessed by ^3^H-methyl-thymidine incorporation assay as previously described [24]. The proliferative response was expressed as stimulation indices (SI, ratio between the mean cpm in stimulated cultures and that in unstimulated cultures). The mean stimulation index in healthy subjects + 3 standard deviations was taken as the threshold level for positivity.

### 2.4. Cytokine Determination

IFN-*γ*, TNF-*α*, IL-4, and IL-10 concentrations in culture supernatants of circulating T lymphocytes were quantified with commercially available enzyme-linked immunosorbent assay (ELISA) sets (OptEIA set, BD Biosciences, CA, USA) as recommended by the manufacturer. The limits of detection were 1 pg/mL for IFN-*γ*, 2 pg/mL for TNF-*α*, IL-4, and IL-10.

### 2.5. Statistical Analysis

Results are expressed as arithmetic means or medians and interquartile ranges. Mann-Whitney *U* and Wilcoxon nonparametric tests were used to investigate the significance of unpaired and paired data. All the covariates were examined in univariate analyses as predictors for actin-specific cellular response. Fisher's exact test and Mann-Whitney *U* test were used to evaluate the differences in discrete and continuous clinical characteristics between patients' groups. A *P* value less than 0.05 was considered statistically significant.

## 3. Results

### 3.1. Proliferative Response of Circulating T Lymphocytes to Actin

**Figure 1:**
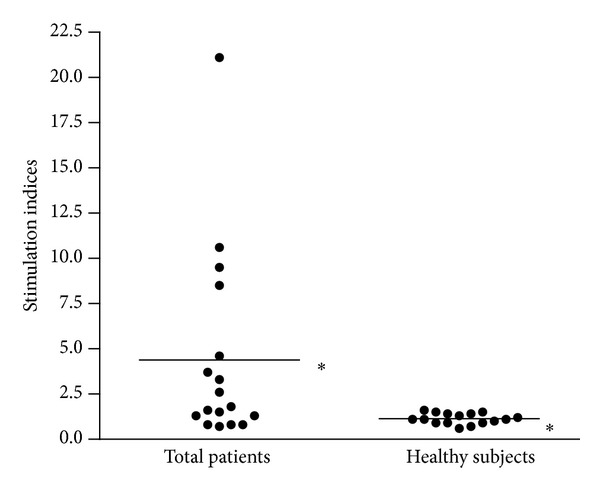
Proliferative response of peripheral blood mononuclear cell samples obtained from the 17 patients with carotid atherosclerosis and from the 16 healthy subjects. *P = 0.02.

**Figure 2:**
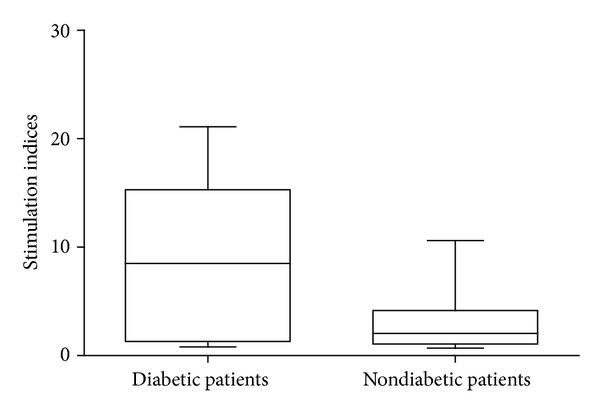
Proliferative response of peripheral blood mononuclear cell samples obtained from the 17 patients with carotid atherosclerosis divided according to the presence/absence of type 2 diabetes. *P = 0.04.

In our selected healthy subject population, we determined a mean SI value of 1.13 and a SD of 0.29 and we calculated the value of 2.0 as the cutoff level for positivity. Actin induced a proliferative response in 8 of 17 (47%) patients' PBMC samples, with SI ranging from 2.6 to 21.1 (Figure 1). PBMC samples from healthy subjects did not proliferate in response to actin. The difference between the SI mean values in patients and healthy subjects was statistically significant (4.4 versus 1.1, *P* = 0.02 by Mann-Whitney *U* test). Univariate analysis showed that the presence of diabetes in patients was significantly associated with proliferative response to actin (*P* = 0.04, Figure 2).

### 3.2. Cytokine Production

**Figure 3:**
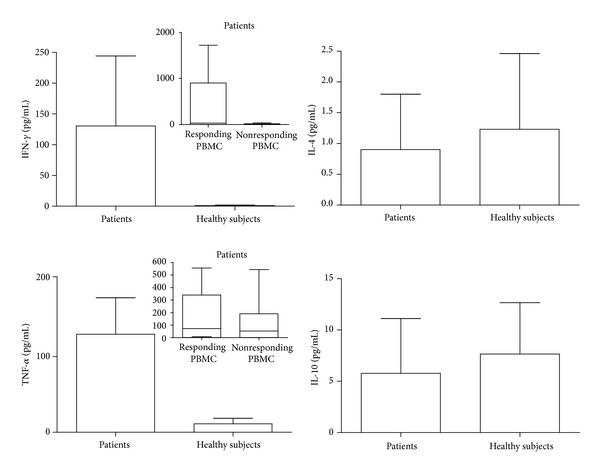
Cytokine secretion in culture supernatants from patients' and healthy subjects' peripheral blood mononuclear cell (PBMC) samples; IFN-*γ* and TNF-*α* production by patients' PBMC responding or not to actin in proliferation assay.

**Figure 4:**
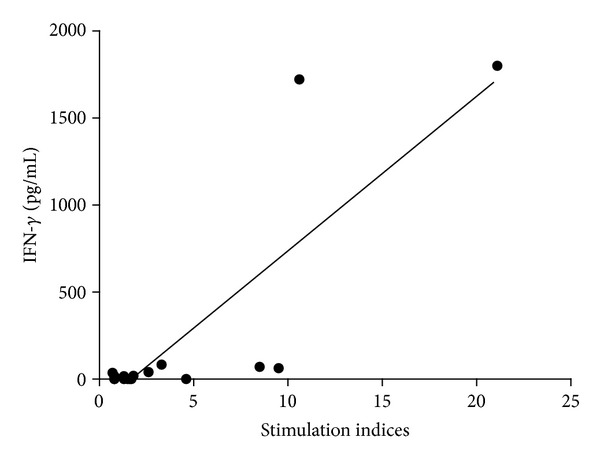
Positive correlation between IFN-*γ* concentrations and proliferative response to actin (stimulation indices) in patients with carotid atherosclerosis. *P* < 10^−4^; *r* = 0.71.

PBMC samples from patients produced higher concentrations of IFN-*γ* and TNF-*α* than PBMC from healthy subjects (Figure 3). In patients, IFN-*γ* and TNF-*α* concentrations were higher in PBMC samples that proliferated in response to actin than in nonproliferating ones (Figure 3). No significant differences were observed for IL-4 and IL-10 production (Figure 3). We found the presence of a positive correlation between IFN-*γ* concentrations and SI (*P* < 10^−4^; *r* = 0.71) (Figure 4). 

## 4. Discussion

In this study, we demonstrated for the first time a role of actin as a target autoantigen of cellular immune reactions in patients with carotid atherosclerosis. As observed for other candidate autoantigens, actin induced a proinflammatory Th1 activation, characterized by high IFN-*γ* and TNF-*α* expression. Th1 response, not counteracted by an increase of anti-inflammatory IL-4 and IL-10 production, may contribute to tissue damage, endothelial dysfunction, and systemic inflammation [12]. Further characterization of these will establish whether they are a regulatory population able to counteract. Our results support previous findings indicating that inflammatory autoimmune reactions are not exclusively localized within atherosclerotic lesions but can also contribute to systemic inflammation in patients with atherosclerosis [12–14]. Inflammation in atherosclerosis is modulated by cytokines that differentially affect endothelial dysfunction. Distinct cytokines promote pro- as well as antiatherogenic processes, thus modulating plaque development and clinical outcome [14, 25, 26]. IFN-*γ* and TNF-*α* mediate proatherogenic processes by promoting monocyte activation and by influencing collagen synthesis and expression of adhesion molecules, tissue factor, and matrix metalloproteinases [27, 28].

Our finding on actin-specific T-cell activation is in line with a previous study where we identified actin as a candidate autoantigen of humoral immune response in patients with carotid atherosclerosis [22]. Actin is a globular protein quite abundant in eukaryotic cells. It can polymerize in the presence of ATP and its structure is remarkably conserved during evolution. Anti-actin antibodies have been associated with various autoimmune diseases including systemic lupus erythematosus [29], a disease in which endothelial damage plays a key role. Anti-filamentous actin antibodies characterize autoimmune hepatitis type 1 where the binding domain of *α*-actinin on actin was shown to be a predominant actin epitope [30]. Anti-actin antibodies were also found in 52–85% of patients with autoimmune hepatitis or chronic active hepatitis, in 22% of patients with primary biliary cirrhosis, and in patients with celiac disease and with autoimmune haemolytic anaemia [31–34]. Furthermore, nonmuscle *α*-actinin 4 and cytoplasmic *β*-actin were identified as immunodominant ovarian autoantigens involved in ovarian autoimmunity [35].

An interesting and extremely important aspect in autoimmune diseases is to understand how abundant and highly conserved self-proteins can become the antigenic target of autoimmune reactions. One of the mechanisms breaking tolerance to self could be apoptosis. It has been shown that apoptotic cancer cells may render actin immunogenic by exposing it on their surfaces [36, 37]. In addition, many autoantigens, and in particular actin, represent a substrate for the proapoptotic cysteine proteases. The polypeptides produced in this way can be released into the extracellular space or can be presented as neoantigens, thus generating an autoimmune response [38, 39]. Interestingly, several studies have shown the presence of apoptotic cells, particularly macrophages and smooth muscle cells, in all stages of atherosclerosis development [40, 41]. 

In our study, we observed a positive association between the presence of diabetes and the response to actin. Pancreatic beta-cell death by apoptosis, which can be induced by multiple stresses, contributes significantly to the pathogenesis of type 2 diabetes [42]. The possibility that diabetes, characterized by increasing oxidative stress and apoptosis, may trigger the autoimmune response to actin is interesting and needs further investigation. 

A limitation of our study is that it does not provide a causal association between the T-cell response to actin and atherosclerosis in humans. *In vivo* experimental models are required to address this question. 

## 5. Conclusions

Our study takes research into the involvement of autoimmune responses in the pathogenesis of atherosclerosis, a small step ahead indicating actin as a candidate autoantigen target of cell-mediated immune responses in a proportion of patients with carotid atherosclerosis. Our findings here call for further studies to identify epitopes on actin recognized by specific T lymphocytes. The identification of these epitopes might be useful to design novel preventive strategies.

## Figures and Tables

**Table 1:** Baseline characteristics of the 17 patients with carotid atherosclerosis.

Parameter	
*N* (%)	17 (100)
Age (years), median (range)	73 (62–84)
Male/female (*n*)	10/7
Diabetes*, *n* (%)	7 (41)
Smoking^†^, *n* (%)	10 (59)
Hypertension^‡^, *n* (%)	10 (59)
Family history^∣∣^, *n* (%)	8 (47)
Hypercholesterolemia^§^, *n* (%)	6 (35)
Body mass index, median (range)	27.7 (25–30.5)
Erythrocyte sedimentation rate, median (range)	15 (12–20)

*Diabetes is type 2, defined as fasting glucose levels ≥140 mg/dL or need for antidiabetic medications.

^†^Smoking is defined as current smokers.

^‡^Hypertension is defined as systolic blood pressure ≥140 mmHg, diastolic blood pressure ≥90 mmHg, or need for hypertension medication.

^∣∣^Family history is defined as having relatives with known heart or vascular disease, including myocardial infarction, heart failure, aneurysm, stroke, sudden death, arrhythmia, and rheumatic fever.

^§^Hypercholesterolemia is defined as total cholesterol >200 mg/dL or need for lipid-lowering therapy.
